# 

**Published:** 1891-07-11

**Authors:** 


PRICE ONE PENNY.
No. 250, Vol. X.—July 11th, 1891.
[Registered at the General Post Office as a Newspaper for transmission in the United Kingdom and Abroad.]

WITH EXTRA NURSING SUPPLEMENT.

Per Annum, 6s. 6d.; post free.

Monthly Parts, 6d.; post free 8d.

Ditto per Annum, 8s., post free.

### THE HOSPITAL

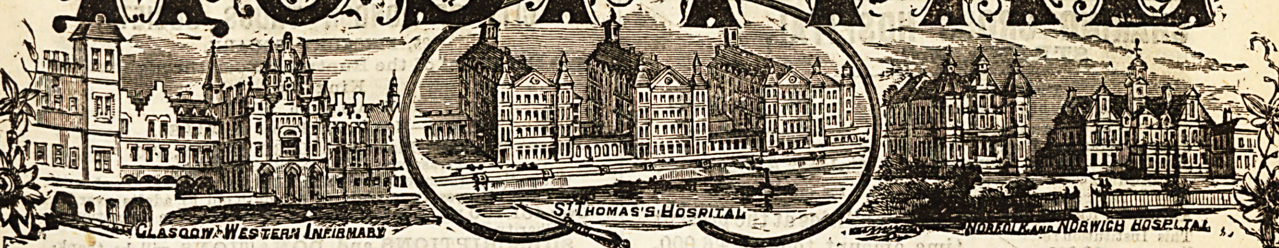

A WEEKLY JOURNAL OF

### Science, Medicine, Nursing and Philanthropy.

### SATURDAY, JULY 11th, 1891.

**The Stress and Strain of Medicine** - 169
**Passing Topics.**—The Programme of the Hospital Saturday Fund - 170
**The Doctor's Armchair.**—"Mad from Life's History" - 171
**Influenza** - 172
**Editor's Letter Box.**—Home for the Dying—The Manchester Chloroform Cases - 173
**Everybody's Page** - 173
**Notes and News** - 174
**General Charities—Scraps and Gleanings** - 175
**Annotations.**—Our Imperial Visitor: A Medical Greeting—Scientific Lunatics—A Call to Intelligent Philanthropists 176
**The Practitioner's Mirror and Retrospect**—
MEDICINE.—The Existence of a Phthisical Contagium, - 177
SURGERY.—Sterno-Mastoid Tumour - 178
THERAPEUTICS.—Cactus Grandiflora - 179
THE HOSPITAL CLINIC.—Swansea Hospital - 179
**The Lords' Committee** - 180
**The Student of Medicine.**—Editorial Notes—Notes from the Schools—Pass Lists—Athletics.

### EXTRA SUPPLEMENT—THE NURSING MIRROR.

**En Passant.**—To Masseurs and Masseuses—The Matron at Putney—Work Abroad—The Princess and the Nurses—Short Items—The First and Second Thousand - lxxxiii
**Lectures on Surgical Ward Work and Nursing.** By ALEX. MILES, M.B. Edin., C.M., F.R.C.S.E.—Lecture XXIX.—Bandages for Lower Extremity - lxxxiv
**Notices** - lxxxiv
**Prize Invalid Jackets** - lxxxv
**For Reading to the Sick**—Fear - lxxxv
**Presentation** - lxxxv
**Asylum Articles.** III.—The Status of Attendants - lxxxvi
**American News** - lxxxvi
**The Sarah Acland Home for Nurses, Oxford** - lxxxvii
**Appointments** - lxxxvii
**Everybody's Opinion.**—The Garden Party—Dewsbury General Infirmary—The Brassey Home - lxxxvii
**Wants and Workers** - lxxxvii
**Notes and Queries** - lxxxvii
**In a Devonshire Village** - lxxxviii
**Amusements and Relaxation** - lxxxvii.

